# Tripartite interactions comprising yeast-endobacteria systems in the gut of vector mosquitoes

**DOI:** 10.3389/fmicb.2023.1157299

**Published:** 2023-06-16

**Authors:** Alessia Cappelli, Claudia Damiani, Aida Capone, Jovana Bozic, Priscilla Mensah, Emanuela Clementi, Roberta Spaccapelo, Guido Favia, Irene Ricci

**Affiliations:** ^1^School of Biosciences and Veterinary Medicine, University of Camerino, CIRM Italian Malaria Network, Camerino, Italy; ^2^Center for Infectious Disease Dynamics and Huck Institutes of the Life Sciences, Department of Entomology, Penn State University, University Park, PA, United States; ^3^Department of Biology and Biotechnology "L. Spallanzani", University of Pavia, Pavia, Italy; ^4^Department of Medicine and Surgery, University of Perugia, CIRM Italian Malaria Network, Functional Genomic Center (C.U.R.Ge.F), Perugia, Italy

**Keywords:** *Wickerhamomyces anomalus*, yeast-endobacteria systems, mosquito, symbionts, vacuole, malaria

## Abstract

It is shown that bacteria use yeast as a niche for survival in stressful conditions, therefore yeasts may act as temporary or permanent bacterial reservoirs. Endobacteria colonise the fungal vacuole of various osmotolerant yeasts which survive and multiply in sugar-rich sources such as plant nectars. Nectar-associated yeasts are present even in the digestive system of insects and often establish mutualistic symbioses with both hosts. Research on insect microbial symbioses is increasing but bacterial-fungal interactions are yet unexplored. Here, we have focused on the endobacteria of *Wickerhamomyces anomalus* (formerly *Pichia anomala* and *Candida pelliculosa*), an osmotolerant yeast associated with sugar sources and the insect gut*.* Symbiotic strains of *W. anomalus* influence larval development and contribute digestive processes in adults, in addition to exerting wide antimicrobial properties for host defence in diverse insects including mosquitoes. Antiplasmodial effects of *W. anomalus* have been shown in the gut of the female malaria vector mosquito *Anopheles stephensi*. This discovery highlights the potential of utilizing yeast as a promising tool for symbiotic control of mosquito-borne diseases. In the present study, we have carried out a large Next Generation Sequencing (NGS) metagenomics analysis including *W. anomalus* strains associated with vector mosquitoes *Anopheles*, *Aedes* and *Culex*, which has highlighted wide and heterogeneous EB communities in yeast. Furthermore, we have disclosed a Matryoshka-like association in the gut of *A stephensi* that comprises different EB in the strain of *W. anomalus Wa*F17.12. Our investigations started with the localization of fast-moving bacteria-like bodies within the yeast vacuole of *Wa*F17.12. Additional microscopy analyses have validated the presence of alive intravacuolar bacteria and 16S rDNA libraries from *Wa*F17.12 have identified a few bacterial targets. Some of these EB have been isolated and tested for lytic properties and capability to re-infect the yeast cell. Moreover, a selective competence to enter yeast cell has been shown comparing different bacteria. We suggested possible tripartite interactions among EB, *W. anomalus* and the host, opening new knowledge on the vector biology.

## 1. Introduction

Bacterial-fungal interactions (BFIs) are known to be generated upon microbial signals of quorum sensing molecules which play roles in a cross-kingdom talk with plant and animal systems (Deveau et al. 2018). Regarding animals, the gut flora of vertebrates and invertebrates is the greatest example of symbiotic association among bacteria, fungi, and hosts, and studying intestinal tripartite relationships may generate truly impactful knowledge. Studies on the intestinal microbiota of diverse animals including insects are increasing, and several bacteria and yeasts have been reported as intestinal symbionts of mosquitoes (Ricci et al. 2012; Cappelli et al. 2021a) but very little is known about BFIs and their impact on insect biology.

Fungal symbiosis with insects and plants is an important evolutionary event with nectar-associated yeasts inevitably entering the digestive system of insects (Witzgall et al. 2012). Nourishing on plant sugars Saccharomycetes (Ascomycota) stimulate plant metabolism and exert antimicrobial properties inhibiting phytopathogens (Lachance and Walker 2018). Insects that feed on plants nectars, therefore, represent an additional symbiotic niche for yeasts.

Interestingly, sugar-rich environments with low water activity like nectars are considered to be stressful for bacteria due to osmotic shock (Lievens et al. 2015), whereas osmotolerant yeasts which show maximum fitness in high concentrations of sugars, become established as normal microflora of such matrices (Pozo et al. 2012). If the situation demands osmotolerant yeasts can acquire bacteria as temporary or permanent residents, and as a result, while seeking shelter, endobacteria (EB) may in turn act as beneficial guests (Siavoshi et al. 2020). EB colonise the yeast vacuole which is an acidic storage compartment similar to mammalian lysosomes and plays a role in biosynthetic pathways and in endocytosis (Thumm 2000). Thus, the yeast vacuole can be considered a specialised niche that enables bacteria to survive in osmotically hostile environments (Siavoshi et al. 2020). For example, yeasts such as *Wickerhamomyces anomalus*, *Candida parapsilosis* and *Meyerozima guillermondii* isolated from sugar-rich matrices (e.g., fruits and commercial foods) can harbour *Staphylococcus* spp. and *Helicobacter pylori*, acting as bacterial carriers and representing an additional route of pathogens transmission to human beyond the close contact among individuals (Siavoshi et al. 2020). This role has been already reported for the Saccharomycetes *Candida albicans*, which harbours endosymbiotic *H. pylori* in the vacuole, providing protection to the bacterium against stresses and antibiotics (Siavoshi and Saniee 2014; Hiengrach et al. 2022).

There are still few reports about yeast-EB systems and in depth studies are needed to assert the biological relevance of BFIs. Moreover, certain Saccharomycetes are utilised in the food industry and have been suggested for biotechnological applications in the environment (Passoth et al. 2011). For instance, *W. anomalus* strains are used as anti-spoilage agents, lethal to bacteria and moulds, for the preservation of alimentary products (Kurtzman 2011). This application of *W. anomalus* considers its broad antimicrobial activity which is based on the secretion of effective killer toxins (KTs) with a β-1,3-glucanase activity (Walker 2011).

Interestingly, symbiotic strains of *W. anomalus* with killer properties have been identified in diverse insects including different mosquito species and a KT-producing strain of *W. anomalus* (*Wa*F17.12) has been isolated from the gut of female mosquito *Anopheles stephensi* (Valzano et al. 2016). *Wa*F17.12 is able to kill sporogonic stages of the malaria parasite *Plasmodium berghei* by the enzymatic effect of a KT and stimulation of the host immune system in *An. stephensi* (Cappelli et al. 2019; Cecarini et al. 2019). On this basis, *W. anomalus* strains residing in the mosquito gut might be exploited as tools for symbiotic control (SC) of mosquito-borne diseases (MBDs) (Cappelli et al. 2021b).

In the present study, we carried out a comprehensive next generation sequencing (NGS) analysis, examining a total of no. 19 yeast samples, to uncover hidden endofungal bacteria and enhance our understanding of yeast as a bacterial reservoir. Specifically, we have explored EB communities of no. 10 *W. Anomalus* strains associated with different mosquitoes such as *Anopheles stephensi*, *An. gambiae*, *Aedes albopictus*, *Ae. aegypti* and *Culex pipiens* which are known vectors of MBDs (i.e., malaria, dengue, etc.). Our research primarily focused on the strain *Wa*F17.12 which has been proposed for the SC of malaria (Cappelli et al. 2021a), with the aim of identifying BFIs that might potentially establish tripartite interactions in the mosquito gut, impacting mosquito biology and/or vector competence. We have disclosed Matryoshka-like associations that comprises *W. anomalus* -EB systems in the gut of the malaria vector *An. stephensi*.

## 2. Results

### 2.1. Metagenomics analyses of *Wickerhamomyces anomalus* strains from diverse sources

**Figure 1:**
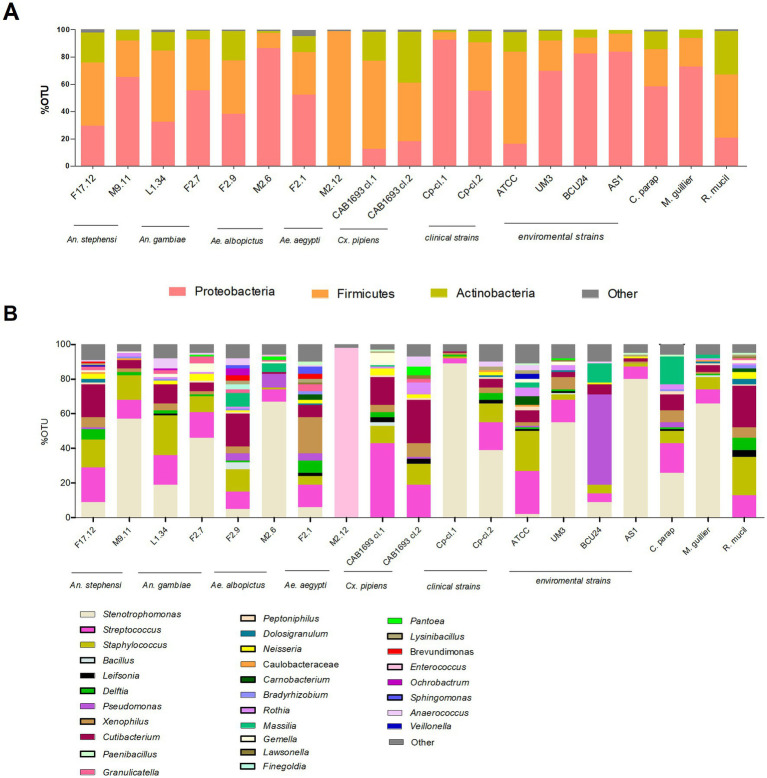
Metagenomics analysis by NGS profiling *16S rRNA* gene of yeast samples listed in Table 1. Stacked bar plots showing the relative abundances of bacterial taxa reads **(A)** at phylum and **(B)** genus level distributed among yeast isolates. *X*-axis indicates yeast strains and *Y*-axis indicates the relative abundance of bacterial taxa calculated as percentage of operational taxonomic units (OTUs). Only OTUs representing >1% of the total reads are represented. The plots are supported with legends showing the yeast strains, bacterial taxa as well as environmental sources of yeast isolates.

A large NGS screening of *16S rRNA* gene profile was conducted encompassing a total of no. 19 yeast samples (Table 1). Ten strains of *W. anomalus* from diverse vector mosquito species (*An. stephensi*, *An. gambiae*, *Ae. albopictus*, *Ae. aegypti* and *Cx. pipiens*), no. 4 *W. anomalus* strains from environmental matrices, no. 2 *W. anomalus* clinical isolates and no. 3 additional yeasts as outgroups (*Candida parapsilosis*, *Meyerozyma guilliermondii* and *R. mucilaginosa*) were analysed (Figure 1). The microbiome sequencing yielded a total of 704.074 reads, varying across samples (minimum = 22.282 maximum = 41.544), with an average of 33.530 reads. Analysis of the rarefaction curves indicated an adequate sampling quality, suggesting a coherent number of sequences reads per sample. The metagenomic analysis revealed numerous operational taxonomic units (OTUs) sharing homology with bacteria from diverse phyla and genera. The most abundant phyla calculated as percentage of OTUs were Proteobacteria (18%–92%), Firmicutes (6%–99%) and Actinobacteria (2%–38%) (Figure 1A). The most abundant genera were *Stenotrophomonas* (6%–89%), *Pseudomonas* (1%–52%), *Xenophilus* (1%–21%), *Delftia* (1%–7%), *Pantea* (1%–5%) and *Sphingomonas* (2%–4%) (Proteobacteria); *Enterococcus* (1%–98%), *Streptococcus* (5%–43%) and *Staphylococcus* (3%–23%) (Firmicutes); *Cutibacterium* (1%–25%) and *Leifsonia* (1%–3%) (Actinobacteria) (Figure 1B). The bacterial frequency among samples was: *Cutibacterium*, *Streptococcus* and *Staphylococcus* (18/19), *Stenotrophomonas* (15/19), *Xenophilus* (13/19), *Delftia* (11/19), *Leifsonia* (8/19), *Pseudomonas* (7/19), *Pantoea* (4/19), *Enterococcus* and *Sphingomonas* (2/19) (Figure 1B). The Principal Coordinates Analysis (PCoA) plots did not visualise trends between groups in our dataset (Supplementary Figure S1).

**Table 1:** Yeast isolates harbouring intravacuolar fast moving BLBs.

Isolates	Yeast species	Source	References
1. *Wa*F17.12	*W. anomalus*	*An. stephensi* (female gut)	Ricci et al. (2011a)
2. *Wa*M9.11	*W. anomalus*	*An. stephensi* (male)	Ricci et al. (2011a)
3. *Wa*L1.34	*W. anomalus*	*An. gambiae* (larvae)	This work/OQ359962^a^
4. *Wa*F2.7	*W. anomalus*	*An. gambiae* (female gut)	This work/OQ359963^a^
5. *Wa*F2.9	*W. anomalus*	*Ae. albopictus* (female gut)	This work/OQ359966^a^
6. *Wa*M2.6	*W. anomalus*	*Ae. albopictus* (male)	This work/OQ359967^a^
7. *Wa*F2.1	*W. anomalus*	*Ae. aegypti* (female gut)	This work/OQ359964^a^
8. *Wa*M2.12	*W. anomalus*	*Ae. aegypti* (male)	This work/OQ359965^a^
9. CAB1693 cl.1	*W. anomalus*	*Cx. pipiens* (larvae)	Steyn et al. (2016)
10. CAB1693 cl.2	*W. anomalus*	*Cx. pipiens* (larvae)	Steyn et al. (2016)
11. *Wa*ATCC	*W. anomalus*	Environment (bread)	Polonelli and Morace (1987)
12. *Wa*UM3	*W. anomalus*	Environment (unknown)	Polonelli et al. (1997)
13. *Wa*BCU24	*W. anomalus*	Environment (olive)	Muccilli et al. (2013)
14. *Wa*AS1	*W. anomalus*	Environment (wine)	Schwentke et al. (2014)
15. *Cp-*cl.1	*W. anomalus*	Clinical isolate	Kalkanci et al. (2010)
16. *Cp-*cl.2	*W. anomalus*	Clinical isolate	Kalkanci et al. 2010
17. *C. parap*	*C. parapsilosis*	*An. gambiae* (female gut)	Bozic et al. (2017)
18. *M. guillie*	*M. guilliermondii*	*An. gambiae* (female gut)	Bozic et al. (2017)
19. *R. mucil*	*R. mucilaginosa*	*An. gambiae* (larval water)	Bozic et al. (2017)

a For the samples identified in the present work (no. 3, 4, 5, 6, 7 and 8) accession numbers are given as reference.

### 2.2. Detection of EB in the vacuole of *Wa*F17.12

**Figure 2:**
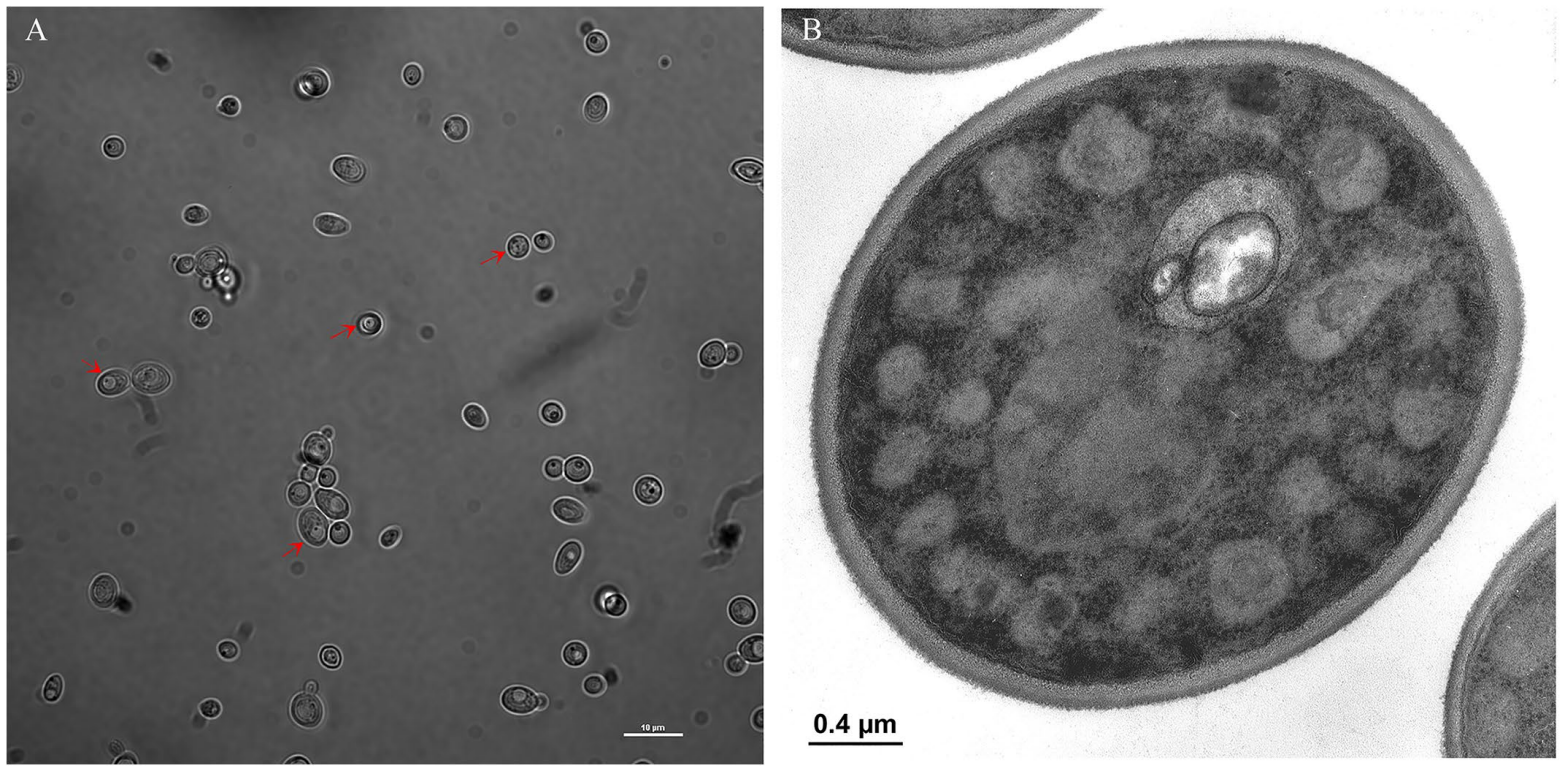
Localisation of EBs in *Wa*F17.12. **(A)** Bright field image at laser scanning confocal microscope (100× objective) shows BLBs (red arrows) in the yeast vacuole (scale bar 10  μm). A video of fast-moving BLBs is available as supplemental material (Supplementary Video S1). **(B)** TEM micrograph of EBs in the vacuole of a *Wa*F17.12 cell. Two EBs with a gram-negative double membrane cell wall surrounded by the yeast vacuolar membrane are visible.

**Figure 3:**
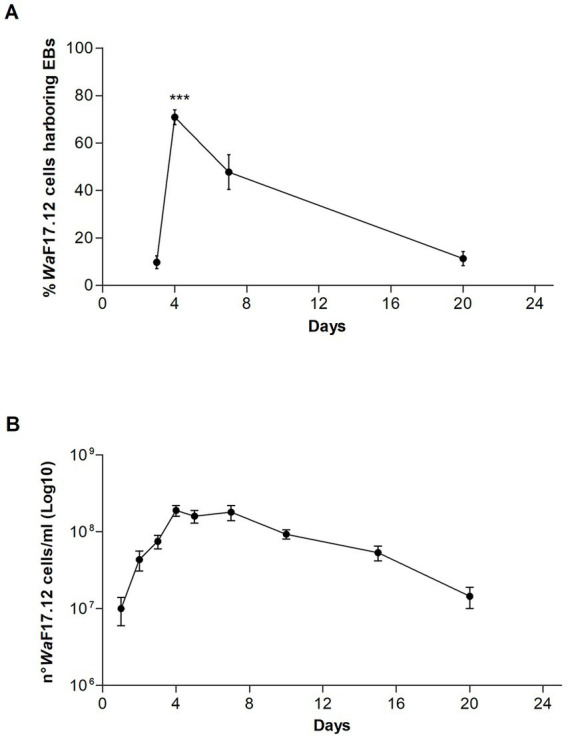
Estimation of EBs in the vacuole of *Wa*F17.12. **(A)** Percentage of *Wa*F17.12 cells harbouring EBs after 3, 4, 7 and 20 culturing days at 30°C in YPD medium with 100 μg/mL ampicillin. Amount of colonised yeast cells has been assessed in optical microscopy with objective 100×. Values have been reported as mean + SEM of triplicate experiments. *p*-value <0.001 (***). **(B)** Growth curve of *Wa*F17.12 analysed in the same culturing condition. The number of yeast cells was evaluated using the trypan blue assay with objective 10×. Values have been reported as mean + SEM of triplicate experiments.

**Figure 4:**
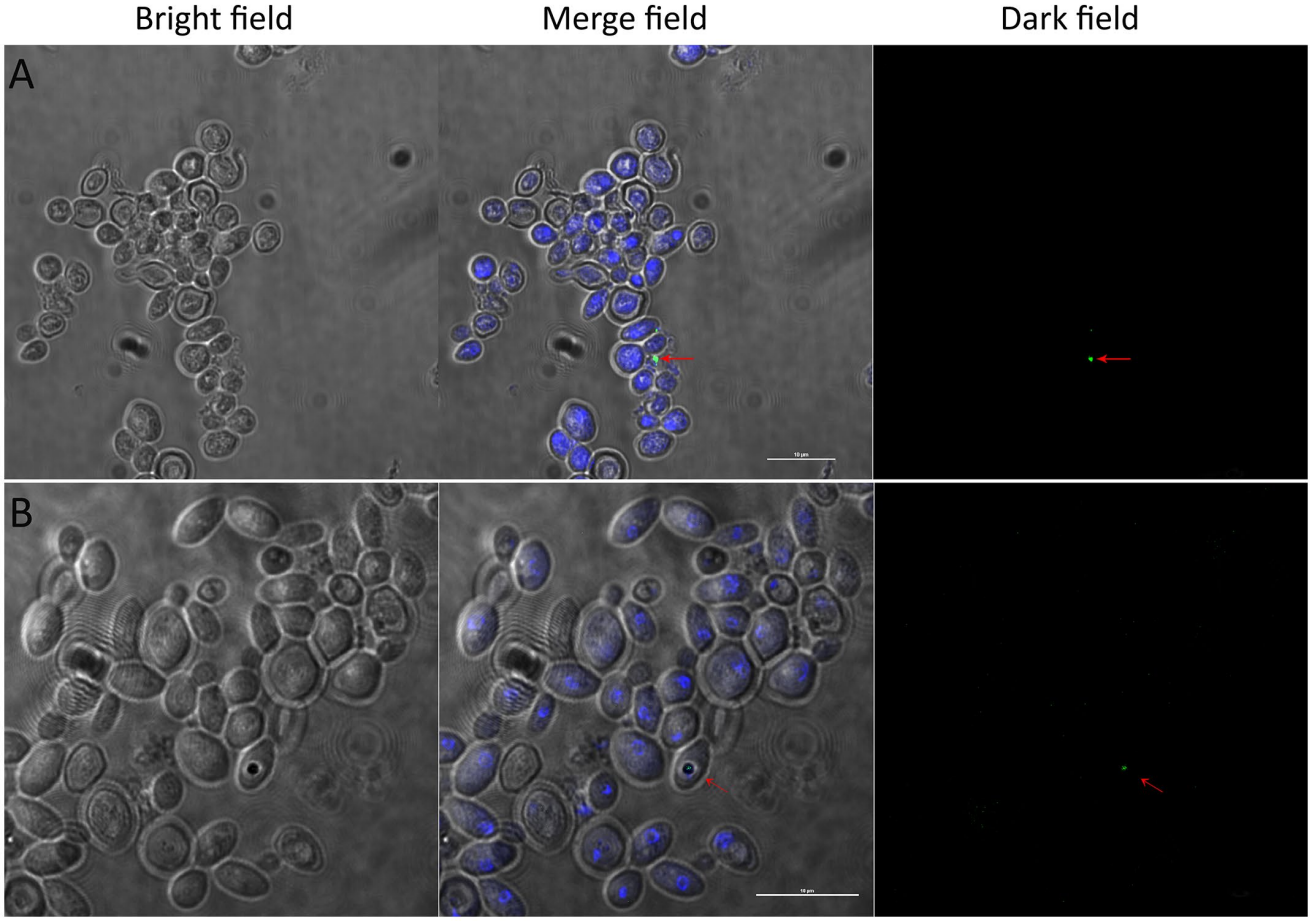
Detection of alive EBs in *Wa*F17.12 in fluorescence assays. Yeast cultures at r.c.p. were observed with a confocal microscope in bright, merged and dark fields (scale bar 10  μm). EBs are visible in merged and dark fields (red arrows). **(A)** FISH assay of *Wa*F17.12 cells with the eubacterial oligonucleotide probe DIG-EUB388; yeast cells (blue, DAPI) and an intracellular bacterium (green, DIG). **(B)** Live/dead-BacLight dye assay of *Wa*F17.12 cells; yeast cells (blue, DAPI) with a viable bacterium (green, Syto) in the yeast vacuole.

We investigated vacuole-bacteria interactions focusing on *Wa*F17.12 which resides in the female gut of the malaria vector *An. stephensi*. Fast-moving bacteria-like bodies (BLBs) were visualised in *Wa*F17.12 using laser scanning confocal microscopy and filmed (Figure 2A ; Supplementary Video S1). The yeast cells were analysed at the ultrastructural level by the means of transmission electron microscopy (TEM), demonstrating the presence of EB surrounded by the yeast vacuolar membrane (Figure 2B). The amount of *Wa*F17.12 cells harbouring intravacuolar EB was analysed at different culturing times (Figure 3). The percentage of colonised yeast cells reached the highest value of 70% on day 4 (reference culturing point = r.c.p. in the present study), EB were still present in 50% of yeast cells after 7 days, gradually decreasing in the following days (Figure 3A). This trend is consistent with the growth curve of *Wa*F17.12 which shows a plateau after 4 days of incubation in culturing conditions (Figure 3B), indicating that new yeast cells inherit EB as part of their vacuolar content. The presence of EB in *Wa*F17.12 was further analysed using fluorescence assays (Figure 4). Eubacterial oligonucleotide probe was used in fluorescence *in situ* hybridization (FISH) of yeast cells to confirm the presence of EB (Figure 4A) and a viability test with a prokaryotic live/dead dye supported the localisation of living bacteria in the yeast vacuole (Figure 4B).

### 2.3. Analyses of bacterial libraries from *Wa*F17.12

To uncover EB associated with *Wa*F17.12, bacterial libraries targeting the *16S rRNA* gene were obtained from different stocks of yeast samples (Supplementary Table S1). Sequence homologies matched to six bacterial targets: *Klebsiella oxytoca*, *Serratia marcescens*, *Enterococcus faecium*, *Staphylococcus warneri*, and *Microbacterium oxydans* and *Leifsonia* sp., revealing a diverse EB population spanning three phyla (Proteobacteria, Firmicutes and Actinobacteria) and five families (Microbacteriaceae, Staphylococcaceae, Enterococcaceae, Enterobacteriaceae and Yersiniaceae) (Table 2). The bacterial phylogenetic trees are provided as supplemental information (Supplementary Figure S2). EB identified in the bacterial libraries included gram-positive strains (*M. oxydans*, *Leifsonia* sp., *S. warneri* and *E. faecium*) and gram-negative strains (*K. oxytoca* and *Se. marcescens*). The molecular identification of gram-negative bacteria in the vacuole of *Wa*F17.12 corroborated previous TEM observations. *Klebsiella*, *Serratia*, and *Microbacterium* were not detected by NGS screenings, likely due to temporary vacuole-bacteria associations (Supplementary Table S1).

**Table 2:** EBs identified in *Wa*F17.12 by screening of bacterial libraries (*) and culture-dependent methods (#).

Phylum; family	Closest relative	GenBank accession No. (% identity)	Project accession No.
Actinobacteria; Microbacteriaceae	*Microbacterium oxidans* ^(* #)^	KT580637.1 (100%)	OQ346104
Actinobacteria; Microbacteriaceae	*Leifsonia* sp.^(* #)^	MH699153.1 (100%)	OQ346105
Firmicutes; Staphylococcaceae	*Staphylococcus warneri* ^(* #)^	MZ674079.1 (100%)	OQ346106
Firmicutes; Enterococcaceae	*Enterococcus faecium* ^(*)^	OQ569372.1 (99.5%)	OQ346107
Proteobacteria; Enterobacteriaceae	*Klebsiella oxytoca* ^(*)^	MT436850.1 (100%)	OQ346108
Proteobacteria; Yersiniaceae	*Serratia marcescens* ^(*)^	MT263018.1 (99.76%)	OQ346109

Partial sequences of the *16S rRNA* gene (778 bp) were analysed using ClustalW and BLAST software. GeneBank accession No. of closest relatives (identity %) are given. Project accession No. are linked to online available data at NCBI website. The phylogenetic tree is reported as supplemental material (Supplementary Figure S1).

### 2.4. Isolation and monitoring of EB from *Wa*F17.12

Isolation of EB was attempted using culture-dependent methods in different stocks of *Wa*F17.12 (Supplementary Table S1). EB were successfully isolated from a few yeast cultures and sequence analyses showed homology to the *16S rRNA* gene of three out of six strains previously identified in the bacterial libraries: *M. oxydans*, *Leifsonia* sp. and *S. warneri*, (Table 2). We hypothesise that the isolation of the other bacteria (*K. oxytoca*, *S. marcescens* and *E. faecium*), may have failed due to limitations of culture-dependent methods. Furthermore, the cultivation of fungal EB might be hindered by the loss of essential genes as in the case of obligate symbionts (Desirò et al. 2014). Specific PCR tests were developed to monitor the association of *M. oxydans*, *Leifsonia* sp. and *S. warneri* in consecutive generations of *Wa*F17.12 cultures (Supplementary Table S1). Bacterial detection was lost after three to four yeast generations, suggesting non-permanent associations with yeast. Furthermore, the bacteria were not found in all the tested samples of our *Wa*F17.12 collection, indicating that the EB composition may vary over time.

### 2.5. Assessment of bacterial lytic activity

**Figure 5:**
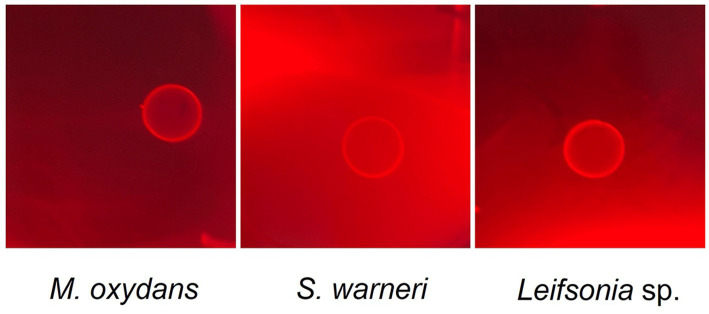
Laminarinase activity of *M. oxydans*, *S. warneri* and *Leifsonia* sp. isolated from *Wa*F17.12. The bacteria were grown on agar plates containing 0.1% laminarin and hydrolysis of the substrate was detected by Congo red staining after 4  days of incubation at 37°C. Clear halos surrounding the growth areas of the bacterial isolates indicate lysis of the polysaccharide.

Further investigations have been carried out to test lytic properties of *M. oxydans*, *Leifsonia* sp. and *S. warneri*. Since a marked laminarinase activity has been reported for *M. oxydans* (Kim et al. 2013), we tested the enzymatic activity of the three bacteria against laminarine (a β-1,3-glucan) (Figure 5). The results supported the occurrence of a bacterial β-1,3-glucanase activity, which targets the same glucan substrates of *W. anomalus* KTs and may interact with antimicrobial activities of *Wa*F17.12.

### 2.6. Re-infection assay of *Wa*F17.12 of with *Staphylococcus warneri* and *Microbacterium oxydans*

**Figure 6:**
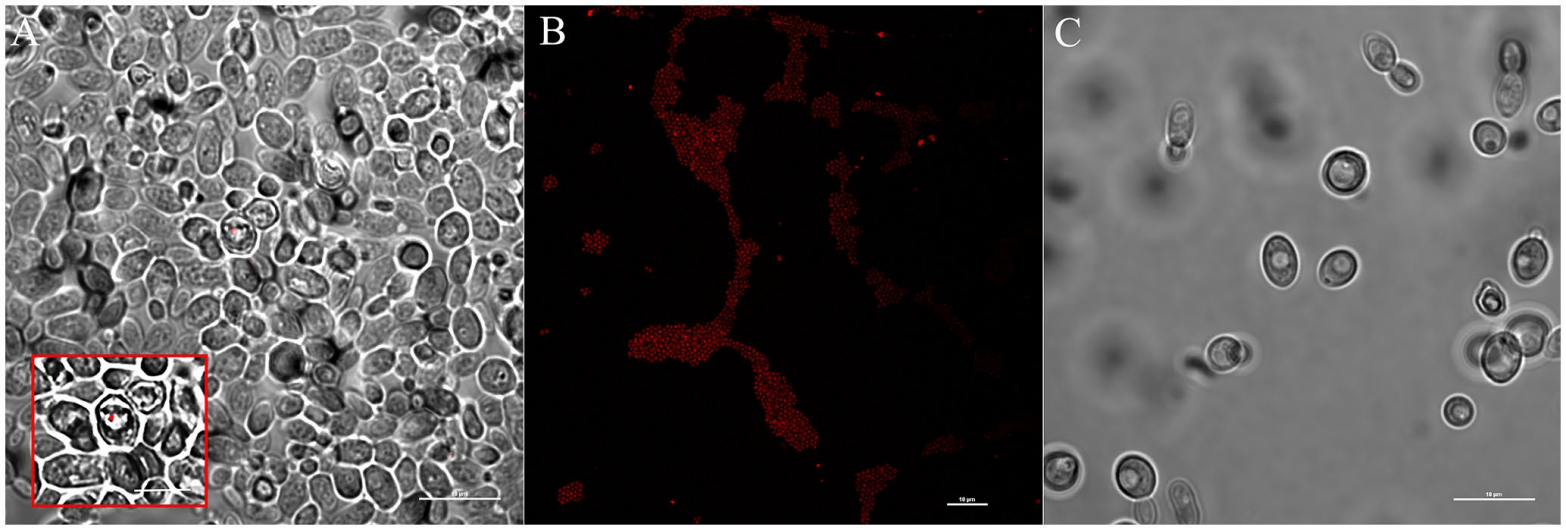
Re-infection test of *Wa*F17.12 with *S. warneri* in FISH assay. Yeast cells (10^6^ cell/mL) co-incubated with bacteria (10^8^ cell/mL) were analysed with the specific bacterial oligonucleotide probe *Swa*-Cy5. Fluorescent red signals labelling bacteria were observed with a confocal microscope and (scale bar 10  μm). **(A)** Bacterial localization of *S. warneri* in the yeast vacuole and magnification (square, scale bar 5  μm); **(B)** *Swa*-Cy5 test in pure culture of *S. warneri*; **(C)** *Wa*F17.12 cultures grown in bacteria-free medium.

To mimic Koch’s postulate, we tested the capability of *S. warneri* and *M. oxydans* to re-infect *WaF17.12*. Bacterial cultures of *S. warneri* or *M. oxydans* were co-incubated with yeast culture and analysed with specific bacterial probes in FISH assays (Figure 6; Supplementary Figure S3, respectively). The results showed the vacuolar localization of bacteria after co-incubation with *Wa*F17.12 cells, whereas fluorescent signals were absent in control yeast cultures grown in bacteria-free medium. We estimated that about one in a hundred yeast cells was colonised by bacteria in the co-incubated cultures. However, the actual re-infection rate was likely higher than estimated due to the technical limitations of oligonucleotide probes to detect intracellular targets localised inside the yeast vacuole.

### 2.7. Comparative invasion test of *Wa*F17.12 with mosquito symbiotic and not symbiotic bacteria

**Figure 7:**
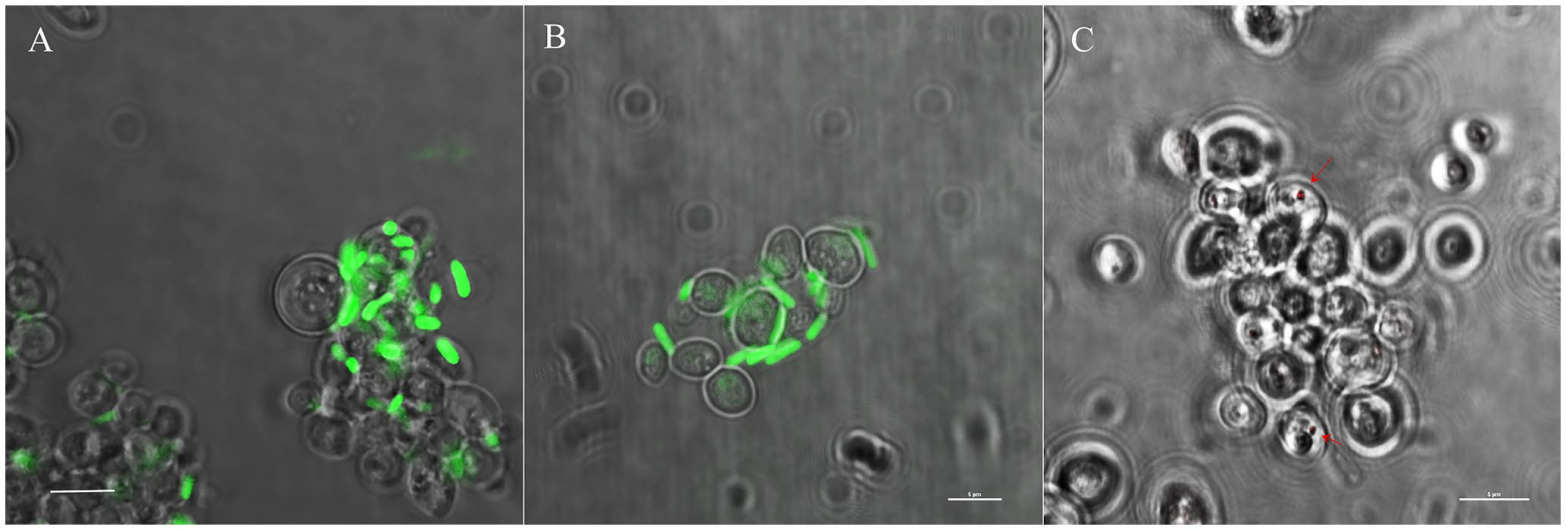
Comparative bacterial infection assay of *Wa*F17.12 cells using fluorescently labelled Serr^AgDsRed^, *Asaia*^SF2.1gfp^ or *E. coli*^gfp^. **(A)** *Asaia*^SF2.1gfp^ and **(B)** *E. coli*^gfp^ (green) are detected outside yeast cells; **(C)** Serr^AgDsRed^ (red) are localized in the yeast cells. Experiments have been carried out in triplicate and samples were visualized after 5  days of co-culturing with yeast using a confocal microscope (scale bar 5  μm).

Diverse symbionts such as *Asaia* (Alphaproteobacteria), *Se. marcescens* (Gammaproteobacteria) and *Wickerhamomyces anomalus* (Ascomycota) have been reported as gut inhabitants in *An. stephensi* (Capone et al. 2013; Cappelli et al. 2014; Adly et al. 2022). On these bases, we conducted an invasion assay of *Wa*F17.12 using fluorescently labelled strains *Serr*^AgDsRed^ (Accoti et al., under review), *Asaia*^SF2.1gfp^ and *Escherichia coli*^gfp^ (Favia et al. 2007). The goal was to compare the ability of mosquito symbiotic bacteria and non-symbiotic bacteria (i.e., *E. coli*) to colonize the yeast cells. *Wa*F17.12 cells were co-incubated with each recombinant strain and observed using confocal microscopy (Figure 7). The results demonstrated that only *Serr*^AgDsRed^ was able to enter the yeast cells, with a colonisation rate of approximately one in twenty-five. This outcome validates the detection of *Se. marcescens* among the EB identified in the bacterial libraries.

## 3. Discussion

Osmotolerant yeasts, such as *W. anomalus*, *C. parapsilosis* and *Me. guillermondii*, which thrive in sugar-rich environments and have been found to harbour intravacuolar EB (Siavoshi et al. 2020), were identified in the mosquito’s gut where they exert metabolic and/or antimicrobial supports (Ricci et al. 2011b; Steyn et al. 2016; Bozic et al. 2017). Of particular interest is *W. anomalus*, which is central to our study. Previous research has demonstrated its association with different mosquito species and its ability to exhibit killer activities against *P. berghei* in the gut of the malaria vector *An. stephensi* (Cappelli et al. 2019). Thus, our comprehensive investigations aimed to (i) identify the EB communities present in mosquito symbiotic strains of *W. anomalus* and (ii) uncover BFIs that might generate tripartite interactions in the mosquito gut, potentially affecting the mosquito biology and/or vector competence.

Through NGS analyses, we have revealed heterogeneous EB communities within *W. anomalus* (no. 10 strains), *C. parapsilosis*, *Me. guillermondii* and *R. mucillaginosa* (no. 1 strain each). This variability is in accordance with metagenomic analysis of *Candida* and *Pichia* species conducted by Indu and collaborators (2021) which highlighted yeasts as thriving bacterial hubs. According to their findings, we detected *Staphylococcus, Streptococcus* and *Cutibacterium* as the most frequent endofungal bacteria (18/19). Interestingly, *Staphylococcus* sp. has been observed previously within the vacuole of *C. albicans* (Tavakolian et al. 2019), while our investigations have not identified *H. pylori*, which is frequently co-detected with *Staphylococcus* spp. in *W. anomalus*, *C. parapsilosis* and *Me. guillermondii* derived from sugar-rich foods (Siavoshi et al. 2020). This suggest a potential preferential association of *H. pylori* with yeast isolates from specific matrices such us fruits and commercial foods (not tested in the present work). Other bacteria that were widely detected in our screening included *Stenotrophomonas*, *Xenophilus*, *Delftia* (15-11/19), and *Leifsonia*, *Pseudomonas*, *Pantoea*, *Enterococcus* and *Sphingomonas* (8-2/19). In particular, we found *Pseudomonas* in *W. anomalus*, *C. parapsilosis*, *Me. guillermondii* and *R. mucilaginosa*, which are yeast species reported as fungal components of the larval mosquito microbiota and are believed to play a role in supporting nutritional functions in the host (Steyn et al. 2016). It would be worthwhile to investigate on BFIs involving *Pseudomonas* and mosquito associated yeasts, as a paradigmatic tripartite interaction has been observed between yeast *Rhodotorula mucilaginosa* and its endosymbiotic bacterium *Pseudomonas stutzeri*. Together, they contribute to nitrogen fixation in the rice plant, that hosts the yeast, thereby stimulating plant growth (Paul et al. 2020).

Regarding the bacterial libraries from *Wa*F17.12 we identified six bacteria (*K. oxytoca*, *Se. marcescens*, *E. faecium*, *S. warneri*, and *M. oxydans* and *Leifsonia* sp.). BFIs were reported in the case of *Microbacterium* sp. and *Se. marcescens*. The former has been observed as an endosymbiont with metabolic functions in *Candida tropicalis* (Kang et al. 2009), while the latter has been reported to invade fungal hyphae of *Mucor irregularis* through prodigiosin, which increases permeability in the fungal cell membrane (Hazarika et al. 2020). We successfully isolated *M. oxidans*, *Leifsonia* sp. and *S. warneri*, demonstrating lytic activities based on laminarinase that might enhance β-1,3-glucanase activity of *Wa*F17.12-KT against the malaria parasite. An “antipathogenic alliance” between EB and fungi has been reported for the endosymbiont *Burkholderia* sp. in the rice plant pathogenic fungus *Rhizopus microsporus*, where the bacterium produces a toxin (e.i. rhizotoxin) that enhances fungal virulence (Partida-Martinez and Hertweck 2005; Valdivia and Heitman 2007; Alabid et al. 2019). However, additional empirical evidence is needed to suggest antimicrobial effects of bacterial laminasinase activities in the mosquito gut. To mimic Koch’s postulate, we also demonstrated through re-infection assays that the three isolated bacteria have the capability to colonise *Wa*F17.12 vacuole. Moreover, using *Serr*^AgDsRed^, a fluorescently labelled strain of *Se. marcescens* isolated from the gut of female *An. gambiae* (Accoti et al., under review), we have demonstrated that the bacterium can penetrate the yeast cell and localise in the vacuole. This outcome support endofungal localisation of *Se. marcescens*, as already described in *M. irregularis*, raising questions about possible tripartite interactions that could impact mosquito biology and/or vector competence. In fact, *Se. marcescens* is known to activate the immune system in female *An. stephensi* impairing *Plasmodium* (Bai et al. 2019).

EB communities uncovered in *Wa*F17.12 and other mosquito associated strains include diverse bacteria that inhabit the mosquito gut. *Se. marcescens* has been found as component of the bacterial microbiota of *Anopheles* spp. (Guégan et al. 2018); *S. warneri*, *M. oxydans* and *Leifsonia* sp. have been reported in *Culex* spp. and *Ae. aegypti* (Pidiyar et al. 2004; Apte-Deshpande et al. 2012; Gunathilaka et al. 2020; Giraud et al. 2022); *Stenotrophomonas maltophilia* and *Xenophilius* have been observed in *An. stephensi* and *Ae. albopictus* (Hughes et al. 2016; Lee et al. 2020); *Kleibsiella* and *Delftia* have been found in *Anopheles* spp. and *Ae. aegypti*, *Pantoea* and *Streptococcus* in *Anopheles* spp. and *Ae. albopictus, Pseudomonas* and *Sphingomonas* in *Anopheles* spp. and *Aedes* spp., *Enterococcus* in *Anopheles* spp., and *Culex* and *Cutibacterium* in *An. gambiae*, *Ae. aegypti* and *Culex* (Guégan et al. 2018). This suggests that that these bacteria may colonise yeast vacuoles during stressful conditions in the mosquito digestive tract such as blood/sugar meal or activation of the host immune system.

In conclusion, based on our findings and the existing literature, the role of yeasts as bacterial reservoirs prompts further inquiry that should be carefully assessed across various environments. The discovered yeast-EB systems provide valuable insights into yeast as bacterial reservoirs. Moreover, the investigations on EB in *Wa*F17.12 has revealed potential tripartite interactions that contribute to a better understanding of mosquito biology. The concept of these Matryoshka-like structures, generated through BFIs in the host gut, introduces new possibilities for utilising microbes against MBDs.

## 4. Materials and methods

### 4.1. Yeast samples and axenic culturing conditions

Nineteen yeast isolates have been included in the present study (Table 1). All the yeast samples were streaked onto YPD (20 g/L peptone, 20 g/L glucose, 10 g/L yeast extract; pH 7) with agar (20 g/L) with rifampicin (40 μg/mL) incubated at 30°C for 3 days (Ricci et al. 2011a). To obtain axenic yeast cultures, purity of single colonies was confirmed under optical microscope with objective 100X (Olympus, Tokyo, Japan). Pure yeast isolates were grown in YPD medium 30°C overnight at 100 rpm and preserved as glycerol stocks (40% v/v) at −80°C.

### 4.2. NGS profiling of *16S rRNA* gene in different yeast isolates

Yeast-endobacteria systems have been analysed by NGS of the bacterial target *16S rRNA* gene in no. 19 yeast isolates (Table 1). Samples included no. 10 *W. anomalus* strains isolated from vector mosquitoes, no. 4 environmental strains of *W. anomalus*, no. 2 clinical strains of *W. anomalus* and no. 3 additional yeast species used as outgroups (*C. parapsilosis*, *Me. guilliermondii* and *R. mucilaginosa*). 16S Miseq analysis was performed on axenic yeast cultures at r.c.p. after observation at a microscope to rule out possible bacterial contaminations. Metagenomic DNAs were extracted from 10^9^ yeast cells per isolate as described above. Yeast-free YPD medium and extraction blanks were included in the analysis as negative controls to evaluate bacterial contaminations occurring before or during the template preparation, respectively. *16S rRNA* gene profiling was conducted by SYNBIOTEC srl (Camerino, Italy). Library preparation was performed by covering the hypervariable region V3-V4 of *16S rRNA* gene using the oligonucleotides 341F and 785R (Klindworth et al. 2013). The data were pre-processed using the Illumina MiSeq—2 × 250 PE—V2 nano, and the reads were sorted by amplicon inline barcodes. No quantifiable libraries were produced from negative controls. Taxonomy was assigned to the reads using Silva Ref *16S rRNA* gene database (Quast et al. 2013). The data presented in the study are deposited in the NCBI repository^1^, accession number PRJNA928690 and in the European Nucleotide Archive, accession number PRJEB60150.

### 4.3. Estimation of BLBs in the vacuole of *Wa*F17.12

For the growth curve assessment, axenic *Wa*F17.12 cultures incubated in YPD medium with 100 μg/mL ampicillin to prevent environmental bacteria contamination, at 30°C in a static way for 20 days have been analysed. The amount of yeast cells was evaluated using the trypan blue assay. Cell counting was achieved in a Neubauer chamber using an optical microscope (Olympus, Tokyo, Japan) with 10× objective. Experiments were carried out in triplicate and values were reported as mean + SEM. The presence of fast moving BLBs in the vacuole of *Wa*F17.12 was monitored after 3, 4, 7 and 20 days of incubation. BLBs were visualised observing a drop of samples at C2+ Laser Scanning Confocal Microscope (Nikon, Tokyo, Japan) with 100× objective. Yeast cells harbouring BLBs were counted in 5 optical fields in triplicate and values were reported as mean percentage + SEM. The highest percentage of yeast cells showing intravacuolar BLBs was observed after 4 days, which has been established as the reference culturing point (r.c.p.) in the present study. The statistical analysis was performed with OneWay ANOVA and Bonferroni *post hoc* test. The movement of BLBs was recorded in a video using the annexed microscope camera.

### 4.4. Transmission electron microscopy of *Wa*F17.12

Axenic *Wa*F17.12 cultures at r.c.p. were washed in 0.9% NaCl, harvested and prefixed in Karnovsky in cacodylate buffer (pH 7.2) (dos Santos and Gregório 2002). After post-fixation in 2% OsO4 for 1.5 h, samples were washed in a cacodylate buffer, dehydrated through an ethanol series, transferred in acetone and embedded in Epon 812. Semi-thin sections were stained with 1% borate methylene blue and examined by light microscope. Thin sections were stained with saturated uranyl acetate, followed by Reynolds lead citrate and examined with a Zeiss EM 900 transmission electron microscope at 80 kV.

### 4.5. Endobacterial viability assay in *Wa*F17.12 and bacterial detection in *Wa*F17.12 By FISH assay

Axenic *Wa*F17.12 cultures at r.c.p. were stained using LIVE/DEAD™ BacLight™ Bacterial Viability Kit (Invitrogen) in accordance with the manufacturer instructions. The method employed a green dye for alive bacteria (Syto) whereas yeast cells were stained using DAPI. Samples were observed with objective 100X using the C2+ Laser Scanning Confocal Microscope (Nikon, Tokyo, Japan). Intravacuolar EB in *Wa*F17.12 cells were detected using the oligonucleotide EUB338 DIG-5′-GCTGCCTCCCGTAGGAGT-3′ which is a universal probe targeting the bacterial *16S rRNA* gene (Indu et al. 2021). *Wa*F17.12 cultures at r.c.p. (2 mL) were centrifuged at 13,000 rpm for 5 min, supernatants were discarded and the cells were fixed in 1 mL of 4% formaldehyde in 1X PBS. Fixed cells were incubated for 3 h at room temperature (rt) and centrifuged. The pellet was suspended in 50% ethanol, incubated for 5 min at rt. and centrifuged. Pellet was washed with 80%, 95% ethanol and air dried in a speed vacuum for 10 min. Hybridisation buffer (20 mM TrisCl, 0.9 M NaCl, 0.01% SDS, 40% formamide) was added to dried cells (500 μL) and incubated at 37°C for 30 min. The probe EUB338 (100 pmol) was added to 50 μL of the pre-hybridisation mixture and incubated for 24 h at 50°C. After 24 h, cells were centrifuged and washed thrice in 0.1× SSC buffer. The mixture was incubated with the antibody anti-DIG conjugated with fluorescein isothiocyanate (FITC) 1:50 in 4% BSA for 30 min and washed three times in 0.1X SSC buffer. The final pellet was dissolved in 20 μL of 0.1× SSC buffer. Slides were prepared and observed with objective 100× under a C2+ Laser Scanning Confocal Microscope (Nikon, Tokyo, Japan).

### 4.6. Bacterial libraries from *Wa*F17.12 and phylogenetic trees

Axenic *Wa*F17.12 cultures at r.c.p. were harvested at 4500 rpm, suspended in a CLB buffer and treated by thermal shock to favour the vacuolar lysis. Genome was extracted using a JetFlex Genomic DNA Purification kit (Invitrogen, Thermo Fisher Scientific, Waltham, MA, United States) according to the manufacturer’s instructions. Partial sequence of the *16S rRNA* gene was amplified using the primers 27F and 805R (Favia et al. 2007). The reactions were carried out in a total volume of 25 μL containing 1x Dream Taq Buffer (Thermo Scientific), 0.25 mM dNTPs, 0.2 mM each oligos, 1 U Dream Taq (Thermo Scientific), amplifying a polymorphic fragment of 778 bp. PCR cycling conditions were: one step at 95°C for 3 min, followed by 30 cycles of a three-step sequence: 95°C for 30 s, 56°C for 30 s, 72°C for 30 s, and a last step at 72°C for 8 min. Amplicons were purified using the DNA Extraction kit (Fermentas, Thermo Scientific) and cloned in JM109 *E. coli* by the pGEM^®^-T Vector System (Promega, Wisconsin, United States) following the manufacturer’s instructions. Subsequently, clones were sequenced and analysed using BLAST and Clustal W software. The 16S gene sequences were deposited through the EMBL-Bank (NCBI) (Accession numbers: *M. oxydans* OQ346104, *Leifsonia* sp. OQ346105, *S. warneri* OQ346106, *E. faecium* OQ346107, *K. oxytoca* OQ346108 and *S. marcescens* OQ346109). Phylogenetic tree of EB from *Wa*F17.12 was performed using MEGA11 (Tamura et al. 2021). The evolutionary history was inferred using the Neighbor-Joining method (Saitou and Nei 1987). The bootstrap consensus tree inferred from 1,000 replicates (Felsenstein 1985) is taken to represent the evolutionary history of the taxa analysed (Felsenstein 1985). Branches corresponding to partitions reproduced in less than 50% bootstrap replicates are collapsed. The evolutionary distances were computed using the p-distance method (Nei and Kumar 2000) and are in the units of the number of base differences per site. The analysis involved 113 nucleotide sequences. All positions containing gaps and missing data were eliminated (complete deletion option). There were a total of 647 positions in the final dataset.

### 4.7. Isolation and identification of EB from *Wa*F17.12

Axenic *Wa*F17.12 cultures at r.c.p. were centrifuged at 13,000 rpm for 5 min, pellets were suspended in a 1X PBS solution with 500 U/mL of Lyticase enzyme (Sigma-Aldrich, Missouri, United States) to break the yeast cell wall and incubated at 37°C for 30 min. This treatment favours the isolation of intracellular bacteria (ref.). Mixtures were plated on LB agar (1% tryptone, 0.5% yeast extract, 1% NaCl, 2% agar; pH = 7) and incubated at 30°C for 4 days. Bacterial colonies were visualised with the 100× objective by optical microscope (Olympus Corporation, Tokyo, Japan) and grown in LB medium overnight at 30°C and 100 rpm. Bacterial cultures (1.5 mL) were pelleted at 13,000 rpm for 5 min and the genome was extracted using a JetFlex Genomic DNA Purification kit (Invitrogen, Thermo Fisher Scientific, Waltham, MA, United States) according to the manufacturer’s instructions. For bacterial identification, the extracted genome was amplified using the universal oligonucleotides 27F5′-AGAGTTTGATCCTGGCTCAG-3′ and 805R 5′-TCGACATCGTTTACGGCGTG-3′ M13-tailed targeting the *16S rRNA* gene and amplifying a polymorphic portion of 778 bp (Favia et al. 2007). PCR reactions were carried out in a total volume of 25 μL containing 1× Dream Taq Buffer (Thermo Fisher Scientific, Massachusetts, United States), 0.25 mM dNTPs, 0.2 mM each oligos, 1 U Dream Taq (Thermo Fisher Scientific, Massachusetts, United States). Cycling conditions were: one step at 95°C for 3 min, followed by 30 cycles of a three-step sequence: 95°C for 30 s, 56°C for 30 s, 72°C for 30 s, and a last step at 72°C for 8 min. Amplicons were purified and sequenced using the M13 oligonucleotides. The sequences were analysed using ClustalW and BLAST softwares and deposited in the EMBL-Bank (NCBI). Accession numbers: *Microbaterium oxydans* OQ346104, *Leifsonia* sp. OQ346105 and *Staphylococcus warneri* OQ346106. Three pure bacterial isolates were preserved as glycerol stocks (40% v/v) at −80°C.

### 4.8. Monitoring *Microbacterium oxydans*, *Leifsonia* sp. and *Staphylococcus warneri* in *Wa*F17.12 with specific PCR tests

Axenic *Wa*F17.12 cultures from yeast collections (stocks of 2016, 2018, 2020, 2021 and 2022 in Supplementary Table S1) were tested in PCR for the presence of *M. oxydans*, *Leifsonia* sp. and *S. warneri* using specific oligonucleotide pairs targeting the *16S rRNA* gene: MoF5′-GAACACGGAGCTTGCTCTGTG-3′ and MoR5′-CGAAATTCTTTCCAGACGCAG-3′ (amplicon 147 bp), LeifF5′-CCACGTACAGGAGATGCCTC-3′ and LeifR5’-AGGGAATTAGTGGCGAACGG-3′ (amplicon 119 bp) (designed in the present work using the NCBI software), SwF5′-TAGTGAAAGGCGGCTTTGCTG-3′ and Sta2R5′-CCGTCAAGATGTGCACAGT-3 (289 amplicon bp) (Gory et al. 1999; Aymerich et al. 2003). Yeast samples were grown in liquid YPD with 100 μg/mL ampicillin for 4 days (r.c.p.) and streaked on plates with 40 μg/mL rifampicin for 24 h, for five consecutive times. Each time three liquid cultures were tested for the presence of the three bacteria with PCR. Yeast cells were centrifuged at 13,000 rpm for 5 min, pellets were suspended in a CLB buffer and incubated thrice in a water bath at 100°C and liquid nitrogen to favour the vacuolar lysis. DNAs were extracted using a JetFlex Genomic DNA Purification kit (Invitrogen, Thermo Fisher Scientific, Waltham, MA, United States) according to the manufacturer’s instructions. PCR reactions were carried out in a total volume of 25 μL containing 1X Dream Taq Buffer (Thermo Scientific, Massachusetts, United States), 0.25 mM dNTPs, 0.2 mM each oligos, 1 U Dream Taq (Thermo Scientific, Massachusetts, United States). Cycling conditions were: one step at 95°C for 3 min, followed by 30 cycles of a three-step sequence: 95°C for 30 s, 58°C for 30 s, 72°C for 30 s, and a last step at 72°C for 8 min. PCR products were run on 1% agarose gel and visualised using the UV light.

### 4.9. Laminarinase activity of *Microbacterium oxydans*, *Leifsonia* sp. and *Staphylococcus warneri*

The three bacterial strains isolated from *Wa*F17.12 were grown in LB plate for 4 days at 30°C, and the laminarinase activity was tested following the protocol described by Kim et al. 2013. A single colony per each strain was transferred with a tube containing the polysaccharide laminarin medium (10 g/L Laminarin, 2 g/L (NH_4_)_2_SO_4_, 0.5 g/L KH_2_PO_4_, 0.1 g/L K2HPO4, 0.1 g/L MgSO_4_, 0.1 g/L NaCl, 0.1 g/L CaCl_2_, 0.5 mL) mineral solution, and 0.5 mL vitamin solution (pH 6.8). After 3 days each bacterial culture (10 μL) was spotted onto laminarin agar plates, and incubated at 37°C for 4 days. To measure the laminarin-degrading ability of each isolate, 0.5% Congo red was poured onto laminarin agar plates for staining. After 15 min, the agar plates were washed twice using 1 M NaCl and clear halos surrounding the growth areas the bacterial isolates indicate were visualised. Experiments have been repeated twice.

### 4.10. *Microbacterium oxydans* and *Staphylococcus warneri* re-infection assay in *Wa*F17.12

Re-infection assays were performed co-incubating pure isolates of *M. oxydans* or *S. warneri* grown in LB medium at 30°C, 90 rpm for 24 h, with three axenic *Wa*F17.12 cultures grown in YPD medium for 48 h. After culturing, yeast and bacterial cells were counted by trypan blue in the Neubauer chamber. Thus, *Wa*F17.12 (10^6^ cell/mL) was co-incubated with each bacterial isolate (10^8^ cells/mL) in YPD medium pH 3 at 30°C/90 rpm for 24 h and then at rt. in a static way for 4 days. Co-cultures with *S. warneri* and *M. oxydans* were analysed by FISH assays as described above using the specific probe *Swa* Cy5-5’-CAGCAAAGCCGCCTTTCAC-3′ (Gory et al. 1999) or *Moxy* FITC-5′-CTGCGTCTGGAAAGAATTTCG-3 (this work). The specificity of the bacterial probes was tested on pure isolates.

### 4.11. Comparative invasion test of *Wa*F17.12 with recombinant bacteria

A comparative invasion assay was performed co-incubating the recombinant strains *Asaia*^SF2.1gfp^, *E. coli*^gfp^ (Favia et al. 2007), and *Serr*^AgDsRed^ (Accoti et al., under review) expressing fluorescent proteins with three axenic *Wa*F17.12 cultures grown in YPD medium for 48 h. *Asaia*^SF2.1gfp^ was grown in GLY medium (yeast extract 1%, glycerol 2.5%, pH 5) with 100 μg/mL kanamycin, *E. coli*^gfp^ and *Serr*^AgDsRed^ were grown in LB medium with 100 μg/mL ampicillin or 20 μg/mL gentamicin at 30°C, 90 rpm for 48 h. Thus, *Wa*F17.12 (10^6^ cell/mL) was co-incubated with each recombinant strain (10^8^ cells/mL) in YPD medium pH 3 at 30°C/90 rpm for 24 h and then at rt. in a static way for 4 days. Co-cultures with fluorescently labelled bacteria were directly observed with a 100× objective under a C2+ Laser Scanning Confocal Microscope (Nikon, Tokyo, Japan).

## Data availability statement

The datasets presented in this study can be found in online repositories. The names of the repository/repositories and accession number(s) can be found below: ENA - PRJEB60150, https://www.ebi.ac.uk/ena/browser/home, NCBI - PRJNA928690, https://www.ncbi.nlm.nih.gov/, OQ346104, https://www.ncbi.nlm.nih.gov/, OQ346105, https://www.ncbi.nlm.nih.gov/, OQ346106, https://www.ncbi.nlm.nih.gov/, OQ346107, https://www.ncbi.nlm.nih.gov/, OQ346108, https://www.ncbi.nlm.nih.gov/, OQ346109, https://www.ncbi.nlm.nih.gov/, OQ359962, https://www.ncbi.nlm.nih.gov/, OQ359963, https://www.ncbi.nlm.nih.gov/, OQ359964, https://www.ncbi.nlm.nih.gov/, OQ359965, https://www.ncbi.nlm.nih.gov/, OQ359966, https://www.ncbi.nlm.nih.gov/, OQ359967.

## Author contributions

IR and AlC: conceptualization. AlC, AiC, and JB: methodology. GF and RS: formal analysis. EC, CD, and JB: investigation. AC and CD: data curation. AlC and AiC: writing—original draft preparation. IR: writing—review and editing, supervision, and funding acquisition. All authors contributed to the article and approved the submitted version.

## Funding

The work was supported by MIUR, PRIN 2020XYBN88_003 to IR.

## Conflict of interest

The authors declare that the research was conducted in the absence of any commercial or financial relationships that could be construed as a potential conflict of interest.

## Publisher’s note

All claims expressed in this article are solely those of the authors and do not necessarily represent those of their affiliated organizations, or those of the publisher, the editors and the reviewers. Any product that may be evaluated in this article, or claim that may be made by its manufacturer, is not guaranteed or endorsed by the publisher.

## Footnotes

^1^ https://dataview.ncbi.nlm.nih.gov/object/PRJNA928690?reviewer=knj5paljed8d12fs98cbaqumd3
