# Supplementary material for: Tripartite interactions comprising yeast-endobacteria systems in the gut of vector mosquitoes
Source: Front Microbiol. 2023 Jun 16;14:1157299. doi: 10.3389/fmicb.2023.1157299 (PMC10311912; doi:10.3389/fmicb.2023.1157299)
Supplement: Supplementary file 1 [file Data_Sheet_1.docx]

Supplementary Material

Tripartite interactions comprising yeast-endobacteria systems in the gut of vector mosquitoes

**Alessia Cappelli^1^, Claudia Damiani^1^, Aida Capone^1^, Jovana Bozic^2^, Priscilla Mensah^1^, Emanuela Clementi^3^, Roberta Spaccapelo^4^, Guido Favia^1^ and Irene Ricci^1*^**

*** Correspondence:** Corresponding Author: [irene.ricci@unicam.it](mailto:irene.ricci@unicam.it)

**Supplementary Tables and Figures**

**Table S1**: EBs identified with culture-dependent and molecular methods in different samples of *Wa*F17.12 collected from 2016 to 2022.

Bacteria associated with yeast cultures for three* or four** consecutive generations. Samples of *Wa*F17.12 have been analysed at r.c.p..

**
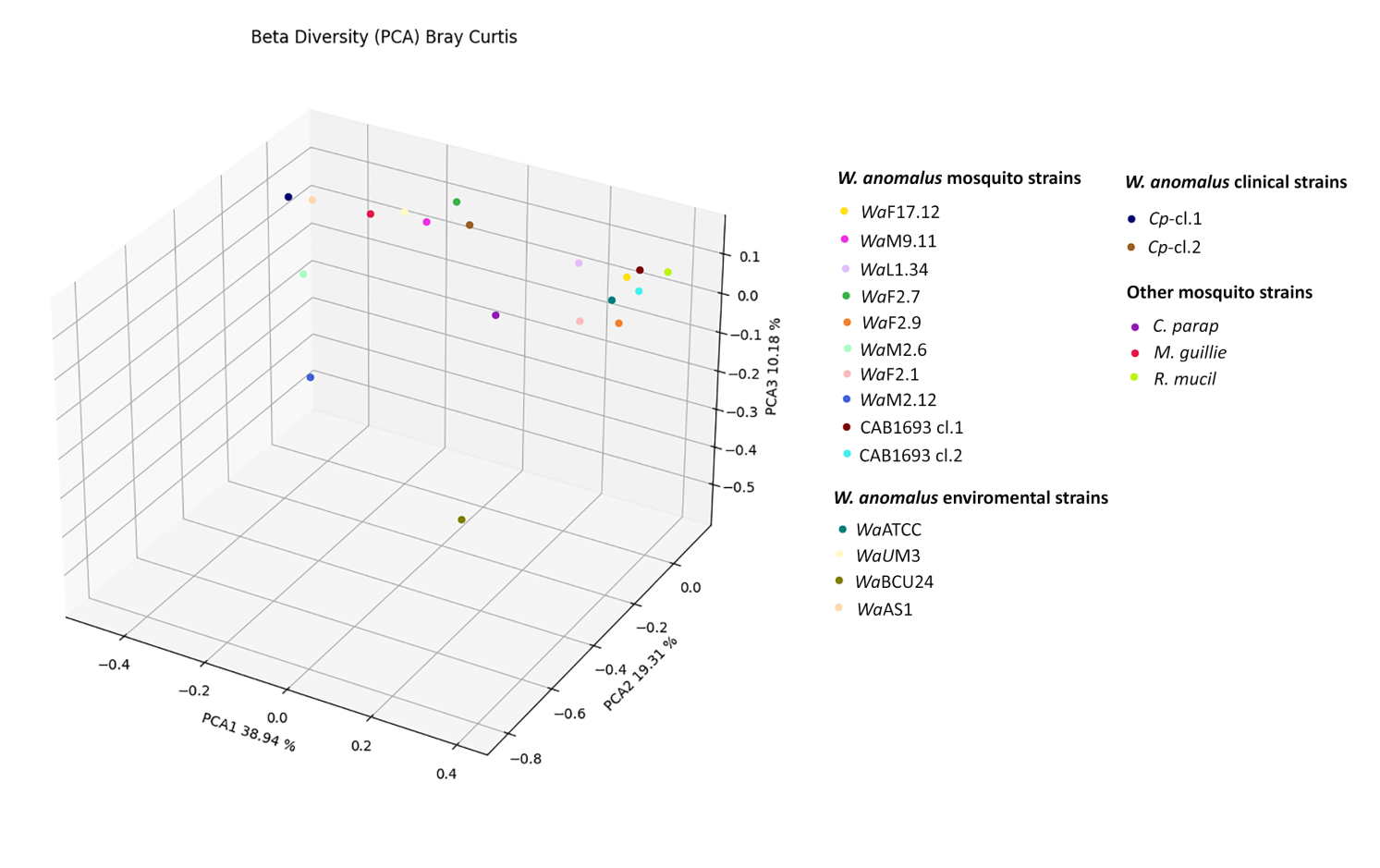
**

**Figure S1.** Principal Coordinates Analysis (PCoA) plots of samples coloured according to different yeast strains. The description of strains is detailed in table 1.


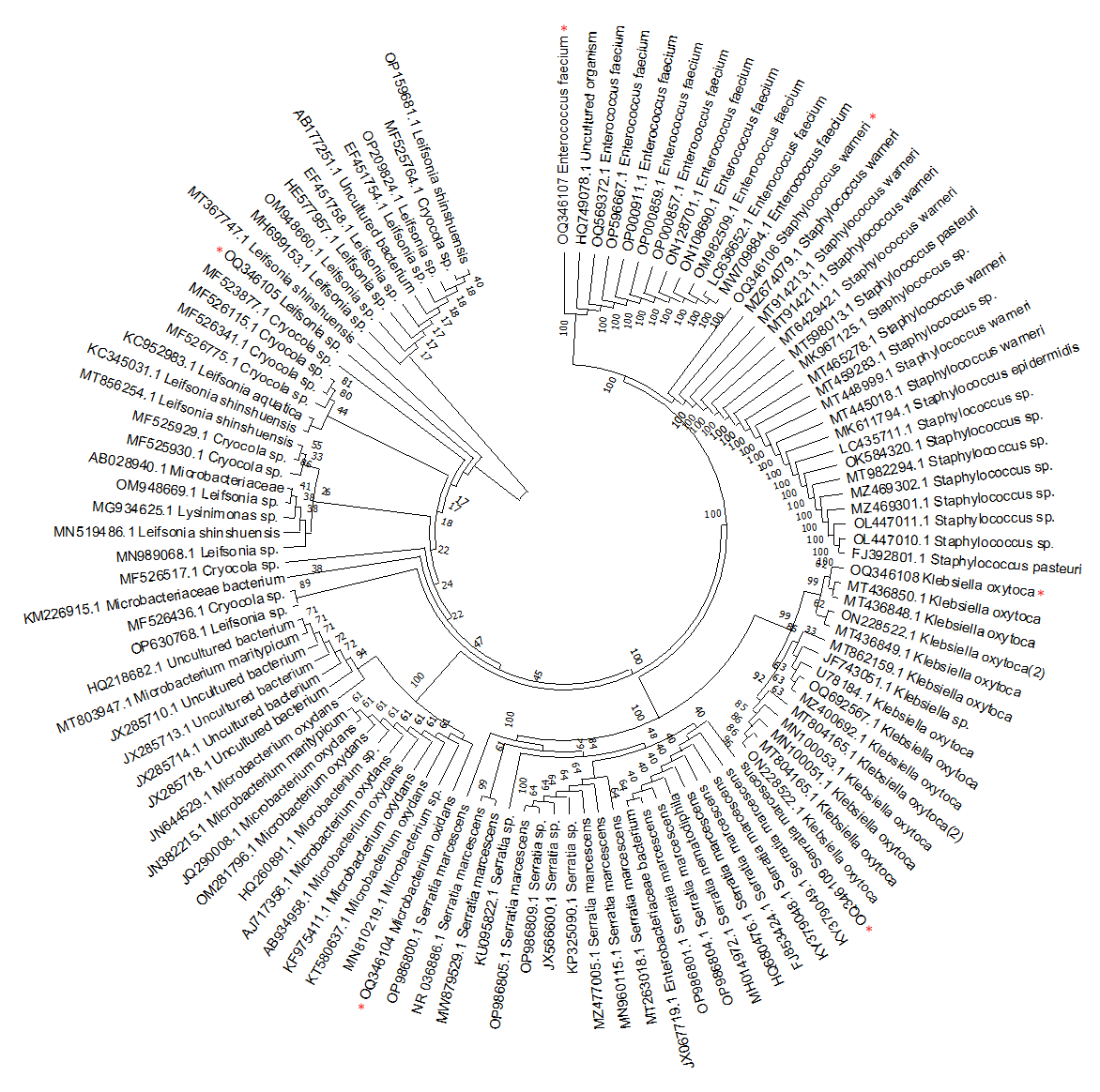


**Figure S2.** Phylogenetic tree of EBs from *Wa*F17.12: *M. oxydans* OQ346104, *Leifsonia* sp.  OQ346105, *S. warneri* OQ346106, *Enterococcus faecium* OQ346107, *Klebsiella oxytoca* OQ346108 and *Serratia marcescens* OQ346109 (red asterisks). The trees were performed using MEGA11 inferred with the Neighbor-Joining method (bootstrap consensus tree from 1000 replicates). Branches corresponding to partitions reproduced in less than 50% bootstrap replicates are collapsed.

**
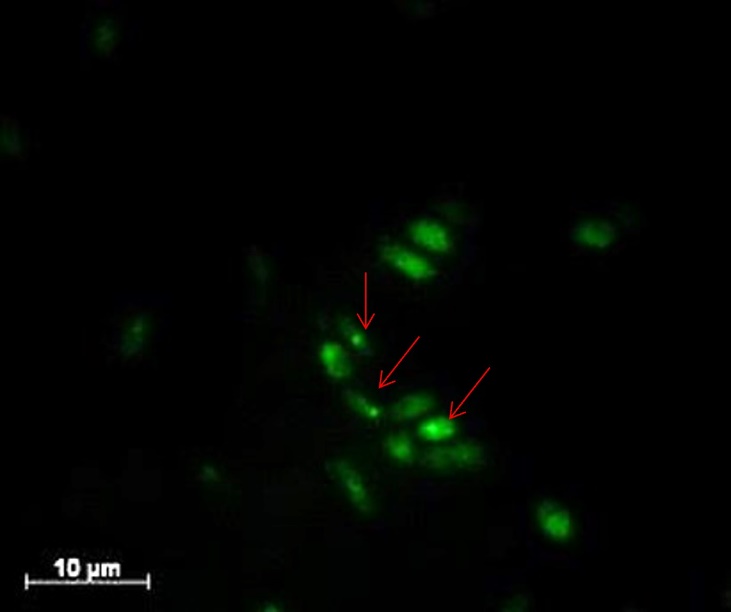
**

**Figure S3.** Re-infection test of *Wa*F17.12 with *M. oxydans* in FISH assay. Yeast cells (10^6^ cell/ml) co-incubated with bacteria (10^8^ cell/ml) were analysed with the specific bacterial oligonucleotide probe *Moxy-*FITC. Fluorescent green signals labelling bacteria in the yeast cells (red arrows) were observed with a confocal microscope and (scale bar 10µm). *Moxy-*FITC was tested in pure culture of *M. oxydans* and *Wa*F17.12 cultures grown in bacteria-free medium were used as control.
